# Production of a Granulysin-Based, Tn-Targeted Cytolytic Immunotoxin Using Pulsed Electric Field Technology

**DOI:** 10.3390/ijms21176165

**Published:** 2020-08-26

**Authors:** Patricia Guerrero-Ochoa, Diederich Aguilar-Machado, Raquel Ibáñez-Pérez, Javier Macías-León, Ramón Hurtado-Guerrero, Javier Raso, Alberto Anel

**Affiliations:** 1 Apoptosis, Immunity and Cancer Group, Aragón Health Research Institute (IIS-Aragón), University of Zaragoza, 50009 Zaragoza, Spain; docenteashlaboratorio@gmail.com (P.G.-O.); rakel_albalate@hotmail.com (R.I.-P.); 2 Food Technology, Facultad de Veterinaria, Instituto Agroalimentario de Aragón-IA2, Universidad de Zaragoza-CITA, 50013 Zaragoza, Spain; diederichaguilarmac@uadec.edu.mx (D.A.-M.); jraso@unizar.es (J.R.); 3 Biocomputation and Physics of Complex Systems Institute (BIFI), University of Zaragoza, 50018 Zaragoza, Spain; jvmacleo@gmail.com (J.M.-L.); rhurtado@bifi.es (R.H.-G.); 4 ARAID Foundation, 50018 Zaragoza, Spain; 5 Department of Cellular and Molecular Medicine, Copenhagen Center for Glycomics, School of Dentistry, University of Copenhagen, 2200 Copenhagen, Denmark; 6 Laboratorio de Microscopías Avanzada (LMA), University of Zaragoza, 50018 Zaragoza, Spain

**Keywords:** granulysin, scFv, immunotoxin, pulsed electric fields, Tn antigen, pancreatic tumors

## Abstract

Granulysin is a protein present in the granules of human cytotoxic T lymphocytes (CTL) and natural killer (NK) cells, with cytolytic activity against microbes and tumors. Previous work demonstrated the therapeutic effect of the intratumoral injection of recombinant granulysin and of the systemic injection of an immunotoxin between granulysin and the anti-carcinoembryonic antigen single-chain Fv antibody fragment MFE23, which were produced in the yeast *Pichia pastoris*. In the present work, we developed a second immunotoxin combining granulysin and the anti-Tn antigen single-chain Fv antibody fragment SM3, that could have a broader application in tumor treatment than our previous immunotoxin. In addition, we optimized a method based on electroporation by pulsed electric field (PEF) to extract the remaining intracellular protein from yeast, augmenting the production and purificiation yield. The immunotoxin specifically recognized the Tn antigen on the cell surface. We also compared the thermal stability and the cytotoxic potential of the extracellular and intracellular immunotoxins on Tn-expressing human cell lines, showing that they were similar. Moreover, the bioactivity of both immunotoxins against several Tn^+^ cell lines was higher than that of granulysin alone.

## 1. Introduction

Granulysin is a protein contained in the granules of cytotoxic T lymphocytes (CTLs) and human natural killer (NK) cells that possesses cytolytic potential on microbes and also on tumor cells [1,2,3]. Our group was the pioneer in the characterization of the molecular mechanism by which recombinant granulysin induces the death of tumor cells [4,5], demonstrating that it fundamentally induces apoptosis in various tumor cells of hematopoietic origin. Later on, we characterized this mechanism in more detail, demonstrating that it was initiated by an increase in cytoplasmic Ca^2+^ concentration, followed by the generation of mitochondrial reactive oxygen species that initiated, in turn, the mitochondrial apoptotic pathway, with caspase 3 activation and nuclear AIF translocation [4,6,7]. In these studies, we also demonstrated that granulysin was able to kill cells from B-cell lymphocytic leukemia patients while being nontoxic against lymphocytes from healthy donors [4]. Recently, we demonstrated the great efficacy of recombinant granulysin in two in vivo tumor development models in nude mice, mammary carcinoma MDA-MB-231 and multiple myeloma NCI-H929 [8]. We also demonstrated that the antitumor action of granulysin was accompanied by a massive infiltration of NK cells in the tumor, providing evidence of immunogenicity, as well as the absence of side effects [8].

In all these experimental approaches, recombinant granulysin was injected intratumorally when the tumor was already detectable. However, considering a possible general clinical application of this treatment, it would be desirable to perform systemic injections of the treatment and, if possible, target granulysin specifically towards the tumor. With this objective, we generated an immunotoxin between granulysin and the anti-carcinoembryonic antigen (CEA) single-chain fraction variable (scFv) antibody fragment MFE23, one of the best approaches to achieve antitumor specificity [9]. We demonstrated that the immunotoxin targeted granulysin against the HeLa-CEA tumor after systemic injection in nude mice xenotransplanted with this tumor. These experiments constitute the proof of concept for the use of these immunotoxins in the systemic treatment of cancer [10].

However, the use of this immunotoxin would be limited to colon or gastric tumors because of the pattern of expression of the CEA antigen. In the present work, we generated a new granulysin-based immunotoxin directed against the Tn antigen. The Tn antigen is overexpressed in a high percentage of tumors of diverse origin, and immunotoxins directed against it would have a broader application. The Tn antigen is formed by a *N*-acetylgalactosamine (GalNAc) residue linked to either serine or threonine residues by means of an alpha-O-glycosidic bond [11]. The SM3 antibody is capable of recognizing the Tn antigen when it is part of a sequence of six amino acids present in the mucin MUC1. Incomplete MUC1 glycosylation in tumor cells leads to the exposure of peptide epitopes, which are masked in healthy cells. The crystal structure of the Tn-glycosylated MUC1 epitope recognized by the antibody SM3 (A1PDT4(a-O-GalNAc)RP6) was characterized recently. Furthermore, glycosylation of Thr produces around a three-fold enhancement in affinity relative to the naked peptide (APDTRP) [12]. In this context, we generated a new immunotoxin between the scFv fragment of SM3 and granulysin.

The SM3 immunotoxin was inserted into a plasmid suitable for production in the yeast *Pichia pastoris*, and its production and purification were optimized in this yeast, which is frequently used in the biotechnology industry to produce recombinant proteins and also certain natural products [13]. However, the amount of protein secreted by yeast to the supernatant was lower than in the case of the other recombinant proteins, and we searched for methods to improve their recovery and purification yield.

Pulsed electric field (PEF) is a technique that consists in the application of an external electric field in order to induce the electroporation of biological membranes. This effect can be reversible or irreversible and leads to a rapid increment of membrane permeability, enabling the release of intracellular biomolecules [14]. In contrast to other extraction techniques, the application of PEF does not result in cell disintegration, minimizing the contamination of the extract with cell debris and facilitating the subsequent purification of the target molecules [15].

In the present work, we used PEF technology to increase the production of the immunotoxin formed by granulysin and the anti-Tn scFv SM3 (SM3GRNLY), comparing the binding to the Tn antigen and the bioactivity on tumoral cell lines of SM3GRNLY obtained from yeast supernatant of yeast submitted to PEF, that is, intracellular SM3GRNLY (iSM3GRNLY).

## 2. Results

### 2.1. Design of Plasmids for the Production of Recombinant SM3-Granulysin Immunotoxin

**Figure 1:**
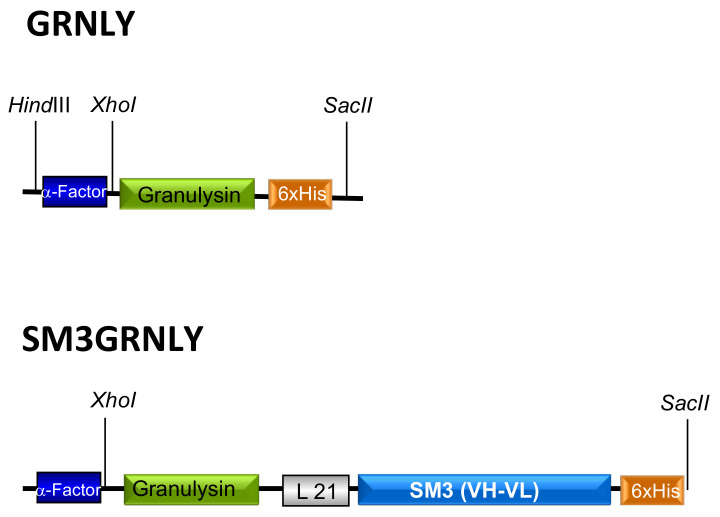
Schematic representations of granulysin (GRNLY) and of the immunotoxin between granulysin and the anti-Tn scFv SM3 (SM3GRNLY). The α-Factor signal peptide is used to direct secretion of the recombinant proteins in *Pichia pastoris*, and the 6xHis tag is appended for immunodetection and affinity purification, respectively.

The construct coding for the 9 kDa granulysin (GRNLY) gene alone was described in our previous work [10]. We designed a construct that will encode for an immunotoxin formed by granulysin bound through a flexible linker of 21 amino acids (formed by Ser and Gly residues) to the anti-Tn scFv SM3 (Figure 1). Both constructs included a tag of six histidines to facilitate subsequent detection and purification. The corresponding plasmids were amplificated in *Escherichia coli* and isolated. Plasmids were then linearized with SacI and purified. Then, plasmids were transfected by electroporation in *Pichia pastoris* and the transfected colonies selected, as indicated in our previous work [10].

### 2.2. Production and Purification of the Recombinant Proteins in Pichia Pastoris. Obtention of the Intracellular Protein by Pulsed Electric Field Technology

Subsequently, GRNLY and SM3-GRNLY were subcloned into the pPICZalphaA vector for production in yeast cells. Both constructs were secreted to the *P. pastoris* supernatant, as demonstrated by Western blot (data not shown). For purification, the extracellular medium of *P. pastoris* cells was collected after 72 h of methanol induction. This time was previously optimized with other recombinan proteins (see [10] and references thereoff), and, in the case of SM3GRNLY, an increase in the time of induction until 96 h or a reduction to 24 or 48 h did not improve the yield of production. Both GRNLY and SM3GRNLY were purified by immobilized metal affinity chromatography, with good yield for GRNLY, around 5 mg/L in the different batches, but with a lower yield for SM3GRNLY, around 2 mg/L. Taking into account the difference in molecular weight, this difference in weight yield meant a difference of 10-fold in molar yield (see Supplementary Table S1). Purified GRNLY exhibited a doublet beween 11 and 12 kDa, in agreement with its predicted molecular weight, taking into account the His-tag and the glycans contribution (see the Coomassie staining of different fractions of purification in Supplementary Figure S1A and the immunoblot analysis of different purified batches using anti-His-tag antibody in Supplementary Figure S1B). Recombinant GRNLY secreted by *Pichia pastoris* has been previously shown to be *O*-glycosylated [16].

On the other hand, SM3GRNLY migrated at a molecular weight around 47 kDa, slightly higher than that calculated from the addition of those of GRNLY and SM3 (see the Coomassie staining of different steps of purification in Supplementary Figure S1C and the immunoblot analysis using anti-His-tag antibody in Supplementary Figure S1D).

In our previous studies, we demonstrated that granulysin was stable at 4 °C in PBS for at least three months [8,10], and, here, we tested SM3GRNLY stability and conservation. We observed that the immunotoxin lost half of its cytotoxic potential against Jurkat cells after one week of storage at 4 °C. Hence, we tested different usual protocols of protein conservation and found that freezing in the presence of glycerol and use immediately after thawing maintained protein bioactivity for months. However, the amount of glycerol used needed to be optimized to avoid toxicity. Although protein stability was guaranteed in the presence of glycerol amounts from 5 to 0.5%, glycerol was toxic by itself in concentrations higher than 1% (see Supplementary Figure S2). Hence, proteins were stored frozen in the presence of 1% glycerol.

**Figure 2:**
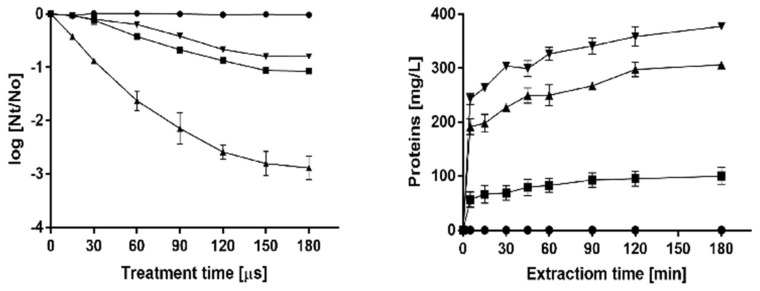
Optimization of intracellular SM3GRNLY (iSM3GRNLY) production from *P. pastoris.* Left panel: inactivation of *Pichia pastoris* by pulsed electric field (PEF) treatment of different electric field strengths. Treatment of 8 kV/cm (●), 12 kV/cm (▼), 16 kV/cm (■), 20 kV/cm (▲). Right panel: effect of different electric field strengths in intracellular protein extraction. Treatment of 12 kV/cm (■), 16 kV/cm (▲), 20 kV/cm (▼) and control (●).

The low yield of immunotoxin production precluded the performance of adequate pre-clinical studies or its future clinical application. This is why we intended the use of pulsed electric fields to improve the yield of production from yeast. The extraction of the immunotoxin that remains in the cytoplasm of the *P. pastoris* cells requires the irreversible electroporation of the cell membrane. As it is well known that irreversible electroporation of cells leads to their inactivation, the number of inactivated cells after the application of PEF was used as an index to estimate treatment conditions required to electroporate the *P. pastoris* cells [17]. The inactivation along the time of *P. pastoris* after exposure to PEF treatments of varying electric field strengths is shown in the left panel of Figure 2. While the treatment applied at 8 kV/cm was ineffective, inactivation was increased with a treatment duration under electric fields of 12 kV/cm or higher. According to these results, the treatment conditions selected to evaluate the release of proteins from *P. pastoris* after electroporation were 12, 16 and 20 kV/cm during 150 µs, corresponding to 84, 90 and 99% of cells inactivated, respectively. Protein release from untreated and PEF-treated cells at different electric field intensities is shown in the right panel of Figure 2. While protein release was not observed in untreated cells, when the cells of *P. pastoris* were previously treated with PEF, the concentration of protein in the medium in which yeast was suspended increased with time. Protein release from the PEF-treated cells increased with the intensity of the applied electric field strength. After 180 min of extraction, the protein concentration in the medium of the *P. pastoris* cells treated at 20 kV/cm was 377 mg/L. This concentration is 3.77 or 1.23 times higher than when the cells were treated at 12 and 16 kV/cm, respectively. According to these results, a PEF treatment of 20 kV/cm for 150 µs and a subsequent extraction period of 180 min were selected for extracting the intracellular SM3GRNLY.

**Figure 3:**
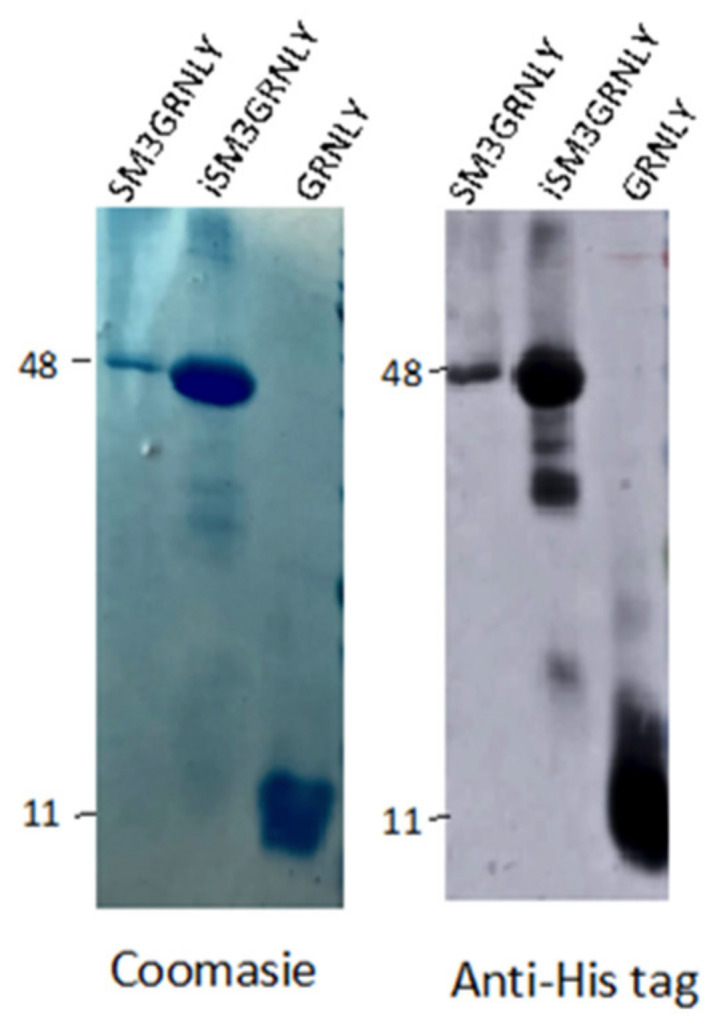
Coomassie staining (left panel) and anti-His tag immunoblot (right panel) of representative purified batches of SM3GRNLY, iSM3GRNLY or GRNLY, as indicated.

As shown in Figure 3, the molecular weight of the purified intracellular protein was very similar to that of secreted SM3GRNLY. In the case of iSM3GRNLY, some degree of protein fragmentation can be observed depending on the batches. However, the 47 kDa band always represented at least a 90% of the total final protein, as quantified by the Image J densitometric program. iSM3GRNLY was stored frozen in the presence of 1% glycerol, as optimized previously with SM3GRNLY (see above).

Hence, we produced SM3GRNLY from yeast supernatant, and we submitted the remaining living yeast to the optimized pulse electric field protocol, recovered the cellular content and purified in this way the SM3GRNLY that had not been secreted previously by yeast (intracellular SM3GRNLY or iSM3GRNLY). An illustration of a representative purification process of iSM3GRNLY is shown in Supplementary Figure S3. Intracellular SM3GRNLY accounted for more than 8 mg per liter of yeast medium, that is, four-fold the protein that was secreted to the supernatant (see Supplementary Table S1). This result indicates that most of the protein was still intracellular (around 80%, depending on the batches).

### 2.3. Assays of Thermal Denaturation

**Figure 4:**
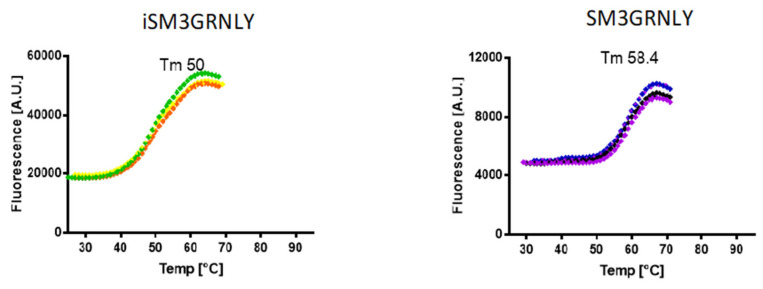
Thermal stability of immunotoxins. SM3GRNLY and iSM3GRNLY stability was tested by differential scanning fluorimetry using Sypro orange dye in the indicated temperature range at pH 7. Triplicate curves are shown and the calculated average temperature (Tm) indicated in each case.

We performed the comparison of the thermal stability of both proteins by differential scanning fluorimetry using Sypro orange dye. As shown in Figure 4, the thermal denaturation curves of SM3GRNLY and iSM3GRNLY were similar, and the melting temperature (Tm) was of 58.4 °C and 50 °C, respectively. This demonstrates that the protein isolated from yeast supernatant was more stable than the protein extracted from yeast using PEF technology, but that both proteins were reasonably stable in the physiological range of temperatures.

### 2.4. Immunotoxin Binding to the Tn Antigen Expressed on the Cell Surface

**Figure 5:**
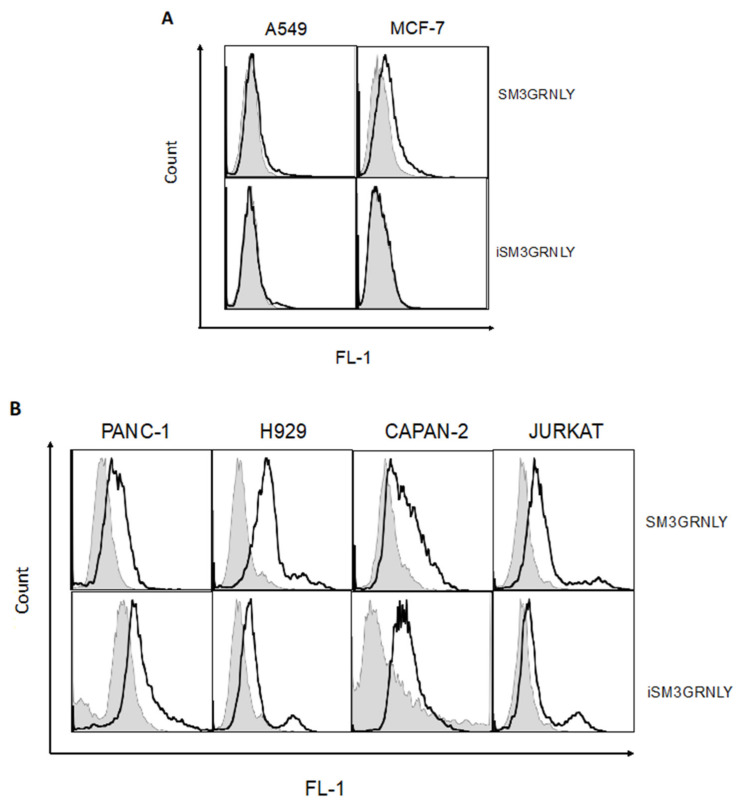
SM3GRNLY and iSM3GRNLY binding to the Tn antigen expressed on the cell surface. (**A**). MCF7 (left panels) and A549 (right panels) were stained with SM3GRNLY (upper panels) or iSM3GRNLY (lower panels), as indicated in Materials and Methods, and labellings analyzed by flow cytometry. Panc-1, Capan-2, NCI-H929 and Jurkat cells were stained with SM3GRNLY or with iSM3GRNLY (**B**), as indicated. Black histograms correspond to the fluorescence of the specific labellings and gray histograms to that of the negative controls.

To determine whether the immunotoxin could bind its antigen on the cell surface, we performed flow cytometry on several tumoral cell lines, as described in our previous study [10]. As shown in Figure 5A, SM3GRNLY (upper panels) and iSM3GRNLY (lower panels) did not substantially bind to A549 lung adenocarcinoma cells or mammary adenocarcinoma MCF7—at least no more than the control reagents. However, SM3GRNLY gave a good Tn labeling on the surface of the two pancreatic adenocarcinoma cells Panc-1 and CAPAN-2, the multiple myeloma cells NCI-H929 and the T cell leukemia Jurkat (Figure 5B), indicating that these tumor cells expressed a high amount of the Tn antigen on their surface and that the SM3 scFv bound to it with high affinity. Of note, we could also demonstrate binding of iSM3GRNLY to the Tn antigen expressed on the surface of Panc-1, Capan-2, H929 and Jurkat cells, albeit at a lower level than SM3GRNLY in the last two cell lines (Figure 5B).

### 2.5. In Vitro Cytotoxic Effect of GRNLY, SM3GRNLY and iSM3GRNLY

**Figure 6:**
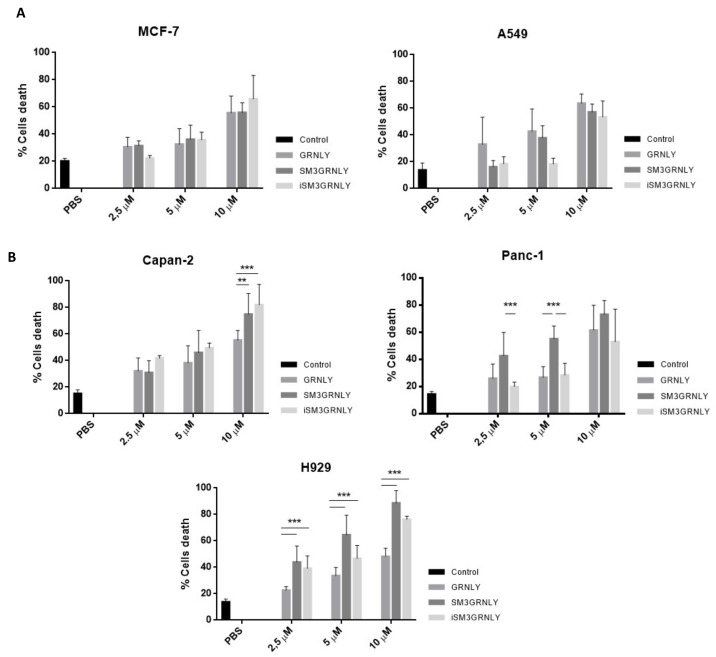
Cytotoxicity of GRNLY and SM3GRNLY on tumoral cell lines. (**A**) A549 or MCF7 cells were incubated with increasing concentrations of recombinant GRNLY, SM3GRNLY or iSM3GRNLY, as indicated, for 24 h. (**B**), NCI-H929, Capan-2 or Panc-1 cells were incubated with increasing concentrations of recombinant GRNLY, SM3GRNLY or iSM3GRNLY, as indicated, for 24 h. Apoptotic cell death was determined in all cases by detection of phosphatidylserine exposure by staining with Annexin-V conjugated with fluorescein isothiocyanate (FITC) and flow cytometry. Basal labelling of cells treated with PBS is also shown as control (black bars). Results are the mean ± SD of at least three different experiments. **, *p* < 0.01; ***, *p* < 0.001.

Next, we addressed if the immunotoxin could promote preferential killing of tumor target cells depending on their level of expression of the Tn antigen or, rather, on the effective binding of SM3GRNLY to their surface. As shown in Figure 6A, the cytotoxicity of SM3GRNLY was the same as that of GRNLY alone on A549 and MCF-7 cells, that is, cell lines that did not show a substantial binding of SM3GRNLY in the flow cytometry experiments shown in Figure 5A. However, the SM3GRNLY immunotoxin exhibited a higher cytotoxicity than granulysin alone on H929 multiple myeloma cells, reducing the IC_50_ from 10 to 2.5 µM (Figure 6B, lower panel). At 10 µM, the percentage of apoptotic cells treated with the immunotoxin was twice that of H929 cells treated with granulysin (90% vs. 45%, respectively). We also demonstrated the enhanced cytotoxicity of the immunotoxin compared with granulysin alone on the pancreatic carcinoma Capan-2 cells, showing that 10 µM of SM3GRNLY induced 70% of cell death, while GRNLY did not induce more than 45% (Figure 6B, left upper panel). Finally, in Panc-1 pancreatic cells, SM3GRNLY showed higher cytotoxicity than GRNLY at low concentrations, but this difference was no longer observed at 10 µM (Figure 6B, right upper panel). Of note, H929, Capan-2 and Panc-1 cells showed a positive labeling when using SM3GRNLY for the flow cytometry analysis shown in Figure 5.

Regarding iSM3GRNLY, we could demonstrate that its cytotoxicity against H929 and Capan-2 cells was similar to that of SM3GRNLY, showing that it was as bioactive as the secreted protein (Figure 6B, light grey bars). In the case of Panc-1, a cell line especially refractory to granulysin toxicity, iSM3GRNLY behaved similarly to GRNLY at all concentrations tested, showing 50% of toxicity at 10 µM concentration.

## 3. Discussion

Our previous studies demonstrate the antitumoral efficiency of recombinant granulysin [4,8]. In addition, we also demonstrated that a chimeric construction composed of granulysin and an anti-CEA scFv maintained, on one hand, its binding to the CEA antigen and, on the other, its bioactivity against tumors [10]. This chimeric immunotoxin was even more cytotoxic against cells that expressed the CEA antigen, demonstrating also its targeting potential, especially in vivo [10]. However, the usefulness of this immunotoxin would be limited to colon or gastric tumors due to the specific pattern of expression of the CEA antigen. We have generated here a new granulysin-based immunotoxin directed against the Tn antigen, which in principle, would have a broader application, since the Tn antigen is overexpressed in a large variety of tumors of diverse origin [11]. In fact, MUC1-associated Tn is one of the first antigens expressed in solid tumors chosen to develop chimeric antigen receptor (CAR) T cells [18,19].

We chose the yeast *P. pastoris* for the production of GRNLY and the immunotoxins because of their high yield of production of folded proteins, disulfide bond formation as well as glycosylation in the absence of endotoxins, frequently associated with recombinant proteins produced in bacteria [20,21]. However, the yield of recombinant SM3GRNLY production in *P. pastoris* was somewhat low. This is why we decided to use pulsed electric field (PEF) technology, a technique that induces the electroporation of biological membranes and the release of intracellular biomolecules [14,22]. In contrast with other extraction techniques, PEF technology does not result in cell disintegration, and in the case of proteins, it does not induce their denaturation [23,24]. The use of PEF for efficient and selective recovery of recombinant proteins such as human ferritin heavy chain (FTH1) or LYTAG-β-galactosidase expressed intracellularly in the yeasts *Hansenula polymorpha* and *Saccharomyces cerevisiae* has been previously reported [23,24]. In these studies, in which electric fields lower than 10 kV/cm and pulsed in the range of milliseconds were used, the subsequent incubation of the permeabilized cells with lyticase was required to obtain high extraction yields. In our case, the use of higher electric fields allowed us to obtain a high efficiency of extraction (4-fold increase) without requiring a following enzymatic treatment, confirming that the technique has a lot of possibilities in the protein biotechnology field.

We first demonstrated that the immunotoxin obtained from yeast supernatant by conventional methods retained its specific binding to the Tn antigen expressed on the surface of different human tumor cell lines. Interestingly, we detected almost no binding of SM3GRNLY to the lung tumor A549 or to the mammary tumor MCF7, although it has been suggested that these cell lines express the MUC-1 Tn antigen. However, in both cases, it was observed that several subpopulations existed with different levels of Tn expression [25,26], and, probably, the level of Tn expression in these cell lines is lower than that in the rest of the cells analyzed. A substantial binding of SM3GRNLY was detected in the acute T cell leukemia Jurkat, in the multiple myeloma NCI-H929, and in the two pancreatic adenocarcinomas Panc-1 and Capan-2. Moreover, the immunotoxin clearly increased granulysin cytotoxicity against these last cell lines, while the cytotoxicity on A549 or MCF7 cells was the same as that observed to granulysin alone. These results suggest that an increased binding of the immunotoxin to Tn expressed on the cell surface correlates with a higher cytotoxicity, and they suggest a positive targeting of the tumoral antigen by the antibody moiety.

Regarding the intracellular protein obtained by PEF technology, we have also shown that it binds to the Tn antigen expressed on the cell surface and, more importantly, that it maintains its cytotoxic bioactivity. iSM3GRNLY showed again a higher cytotoxicity than GRNLY alone, at least on H929 and Capan-2 cells, that was around the same as that of the immunotoxin obtained from yeast supernatant.

Immunotoxins normally combine a bacterial or plant toxin with full-length IgG or to scFv antibody fragments. For example, the recently approved moxetumomab pasudotox combine the PE38 Pseudomonas endotoxin with and anti-CD22 scFv, and it is suitable for the treatment of hairy cell leukemia [27]. However, the immune reaction against the toxin moiety in patients may reduce their clinical success [28,29]. In this respect, the granulysin-based immunotoxins described here or in our previous work [10] are devoid of immunogenicity, since granulysin is a physiologically normal human protein.

In summary, on one hand, the use of PEF technology to obtain the recombinant anti-Tn SM3GRNLY immunotoxin substantially increased its production yield, clearing the way for its future production escalation in biotech companies. On the other hand, the confirmation of the increased cytotoxicity of granulysin-based immunotoxins on tumoral cells that express the targeted antigen, together with their lack of immunogenicity, makes them good candidates as new anti-tumoral agents.

## 4. Materials and Methods

### 4.1. Cell Culture

Human tumor cell lines were cultured at 37 °C and 5% CO_2_ using standard procedures. The acute T cell leukemia Jurkat and the multiple myeloma cells line NCI-H929 were cultured in RPMI 1640 medium supplemented with 10% fetal bovine serum (FBS) (Pan Biotech, Aidenbach, Germany). The mammary adenocarcinoma MCF7, the non-small cell lung cancer A549 and the pancreatic adenocarcinoma cell lines Panc-1 and Capan-2 cultured in DMEM medium (Pan Biotech GmbH) supplemented with 10% FBS (Sigma, Madrid, Spain). Culture media were supplemented with penicillin/streptomycin (Pan Biotech) and GlutaMAX (Invitrogen, Barcelona, Spain), and the absence of mycoplasma was routinely tested by PCR.

### 4.2. Bacterial Strains, Plasmids, and Culture Conditions

The *E. coli* DH5α strain was grown at 37 °C in Luria-Bertani medium (LB; Oxoid, Basingstoke, UK). *Pichia pastoris* was grown at 30 °C in yeast extract with peptone and dextrose (YPD) broth (Formedium) for routine maintenance and in buffered glycerol-complex medium (BMGY) (1% yeast extract, 2% peptone, 1.34% yeast nitrogen base (YNB) 1% glycerol, 400 μg/L biotin, and 0.1 M potassium phosphate, pH 6.0) for expansion and big-scale production, followed by cultured at 18 °C in buffered methanol-complex medium (BMMY ) (1% yeast extract, 2% peptone, 1.34% YNB, 1% methanol, 400 μg/L biotin, and 0.1 M potassium phosphate, pH 6.0) for induction of the recombinant protein. The synthetic gene encoding 6xHis-tagged 9 kDa granulysin was synthesized and inserted in the pPICZαA plasmid as indicated in Ibáñez 2019. The synthetic gene coding for 6xHis-tagged SM3GRNLY was synthesized and inserted in the pPICZαA plasmid by Genscript (Leiden, The Netherlands). Both plasmids were amplificated in *E.coli* and isolated by NucleoSpin^®^ Plasmid EasyPure (Macherey-Nagel). Plasmids were linearized with SacI (Takara) and purified by Ilustra™ GFX™ PCR DNA and Gel Band Purification kit (GE Healthcare). The transformation of *P. pastoris* and the selection of transfected colonies was performed by the methods described in Wiedner 2010 and Ibáñez 2019.

### 4.3. Expression and Purification of Extracellular Recombinant Granulysin and SM3-Granulysin Immunotoxin in Pichia Pastoris

The preinoculum of *P. pastoris* cell strains X33 for SM3GRNLY and SMD1168 for GRNLY, was cultured in 100 mL YPD medium overnight at 30 °C for activation; the cells were then added to 1000 mL of BMGY medium, incubated overnight at 30 °C for growth and finally overnight in BMMY medium at 18 °C for induction. All these culture steps were performed in a thermostated shaking incubator at 250 rpm as shaking intensity. The culture was fed with 1% methanol every 24 h for 2 or 3 days; then, supernatants were concentrated using Pellicon^®^ XL Ultracel 0.005 m^2^ cassettes (MerckMillipore, Burlington, MA, USA), dialyzed in PBS and proteins purified by Ni^2+^ affinity chromatography (Ni-NTA agarose, Qiagen, Madrid, Spain), with an elution buffer containing 20 mM imidazole, 300 mM NaCl and 20 mM Tris-HCl, pH 8. Eventually, the eluate was concentrated and its buffer was changed to PBS using Amicon^®^ filters (MerckMillipore).

### 4.4. Expression and Purification of Intracellular Recombinant SM3-Granulysin Immunotoxin from Pichia Pastoris by Pulsed Electric Field Technology

The PEF equipment used in this work was the commercial model EPULSUS^®^-PM1-10 (Energy Pulse System, Lisbon, Portugal). *P. pastoris* cells were resuspended in McIlvaine buffer (pH 7.0 and 1.50 mS/cm) at a concentration of around 10^8^ cells/mL and PEF-treated in continuous flow (5.0 L/h) in parallel electrode chamber of 3.0 cm length, 0.50 cm width and a gap of 0.40 cm. The calculated mean residence time in the treatment chamber was 0.40 s. A heat exchanger consisting of a coil submerged in a thermostatic batch was used to set the initial temperature of the yeast before the treatment at 10 °C. The temperature of the yeast suspension after the PEF treatment chamber never exceeded 30 °C.

*P. pastoris* cells were PEF treated at electric fields strength between 8 and 20 kV/cm for treatment times between 15 and 180 µs. After the treatments, serial decimal dilutions were poured plated in potato dextrose agar in order to monitor *P. pastoris* cell inactivation. The number of viable cells was expressed in colony-forming units (CFU), corresponding to the number of colonies counted after 48 h of incubation at 25 °C. Inactivation data were expressed as the ratio between the initial number of survivors (No) and the number of survivors after different treatment times (Nt).

Release of protein from untreated and PEF-treated (12, 16 and 20 kV/cm for 15 to 180 µs) cells of *P. pastoris* was monitored after 180 min of incubation at 20 °C in a McIlvaine buffer solution of pH 7 and 1 mS/cm of conductivity. Quantitative analysis of released proteins was conducted by the microplate procedure of Pierce™ BCA Protein Assay Kit (Thermo Scientific, Rockford, IL 61105, USA).

### 4.5. Protein Stability Determinations

Thermal stability tests were performed with the differential scanning fluorimetry technique also known as thermal displacement test (thermofluor). The experiment was performed on a 96-well plate in the Stratagene Mx3005P (Agilent Technologies, Waldbronn, Germany). In a final reaction volume of 40 μL, the proteins, at a final concentration of 0.5 mg/mL, were incubated in the presence of Sypro orange (Invitrogen) at a final 5-fold concentration, following the manufacturer’s instructions. The reaction was performed in 75 cycles of 60 s each, with an initial temperature of 25 °C and an increase of 1 °C per cycle. The emitted fluorescence was captured in each cycle and was plotted on Graphpad prism. To obtain the Tm, the curve data were fitted to the Boltzmann equation. Determinations were performed in 25 mM HEPES pH 7.0.

### 4.6. Flow Cytometry Analysis of Binding of SM3GRNLY or iSM3GRNLY to the Tn Antigen

To analyze binding to the Tn antigen on the surface of living cells, 10^5^ cells per well were placed in a 96-well round-bottom plate. Cells were first incubated with SM3GRNLY or iSM3GRNLY at 10 μg/mL in PBS with 5% FBS for 30 min at 4 °C followed by mouse anti-histidine tag antibody (1:200; Genscript, Leiden, The Netherlands) and goat anti-mouse antibody bound to FITC (1:200; Caltag, Barcelona, Spain). After each incubation, cells were washed with 5% FBS in PBS. The binding was determined on the surface of cells using a FACScalibur flow cytometer (BD Biosciences). Control negative labellings were also established by making the same incubations indicated above, but in the absence of SM3GRNLY or iSM3GRNLY.

### 4.7. Cytotoxicity Assays

Cells were seeded in complete medium at 1 × 10^6^ cells/mL in 50 μL per well in 96 well plates, and GRNLY, SM3GRNLY or iSM3GRNLY were added at the indicated concentrations. In control wells, the same volume of PBS was added in each case. Cells were incubated for 24 h at 37 °C and cell death was analyzed by analyzing PS exposure by flow cytometry using Annexin-V-FITC (BD Biosciences, Madrid. Spain). For this, and after the incubation period, cells were washed with PBS and incubated with Annexin-V-FITC in annexin-binding buffer (140 mM NaCl, 2.5 mM CaCl_2_,10 mM Hepes/NaOH, pH 7.4) for 15 min and analyzed by flow cytometry.

### 4.8. Statistical Analysis

Statistical significance was evaluated by using Student *t*-test for non-paired variants. Differences were considered significant if *p* < 0.05.

## 5. Patents

The use of granulysin immunotoxins as an anti-tumoral treatment is protected by the patent application PCT/ES2018/P201830768 presented to the Spanish Bureau of Patents and Brands (OEPM) on 07/26/2018, extended to international application with the World Intellectual Property Organization (WIPO) publication number WO/2020/020978 on 01/30/2020.

## Supplementary Materials

The following are available online at https://www.mdpi.com/1422-0067/21/17/6165/s1.
