# Supplementary material for: Production of a Granulysin-Based, Tn-Targeted Cytolytic Immunotoxin Using Pulsed Electric Field Technology
Source: Int J Mol Sci. 2020 Aug 26;21(17):6165. doi: 10.3390/ijms21176165 (PMC7503585; doi:10.3390/ijms21176165)
Supplement: Supplementary file 1 [file ijms-21-06165-s001.pdf]

Supplementary material

Supplemental Table 1. Comparison of the yield of production of the three recombinant proteins used in the study.

**Protein**	**Source**	**Yield** (total protein per liter of yeast med	**Molar yield** (nmoles per liter of yeast medium**)**	***n***
GRNLY	Extracellular	5.040 mg	458 nmol	5
SM3GRNLY	Extracellular	2.115 mg	45 nmol	6
iSM3GRNLY	Intracellular	8.260 mg	176 nmol	7

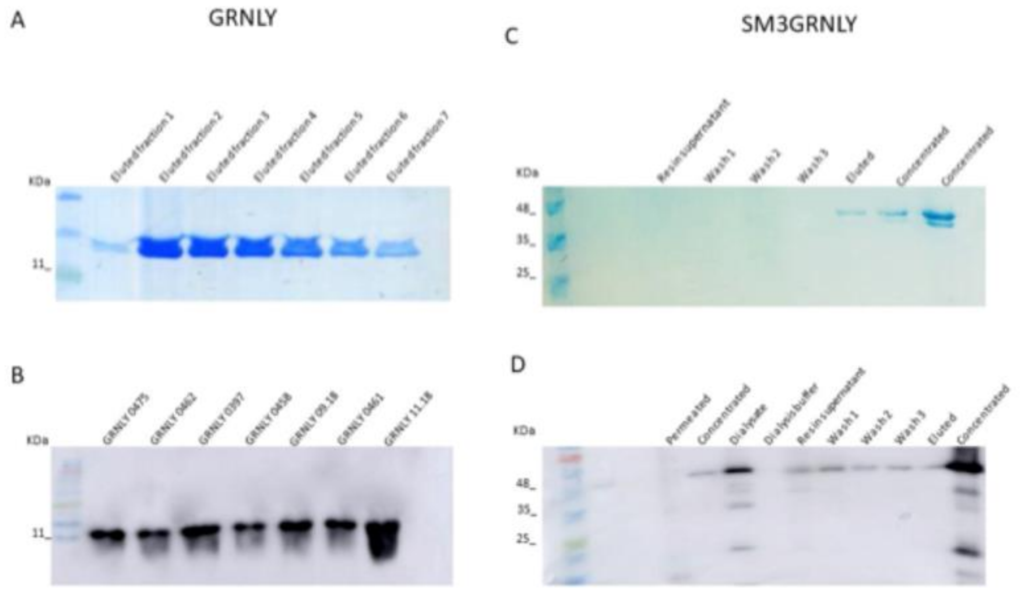

Supplementary Figure 1. Characterization of purified GRNLY and SM3GRNLY. A, C) Coomasie staining of elution fractions from the Ni-NTA-agarose column of a representative GRNLY batch (**A**); or of different steps of the purification process on the Ni-NTA-agarose column of a representative SM3GRNLY batch (**C**). (**B,D**) Anti-His inmmunoblot of different purified batches of GRNLY (**B**); or of different steps in the purification process of a representative SM3GRNLY batch.

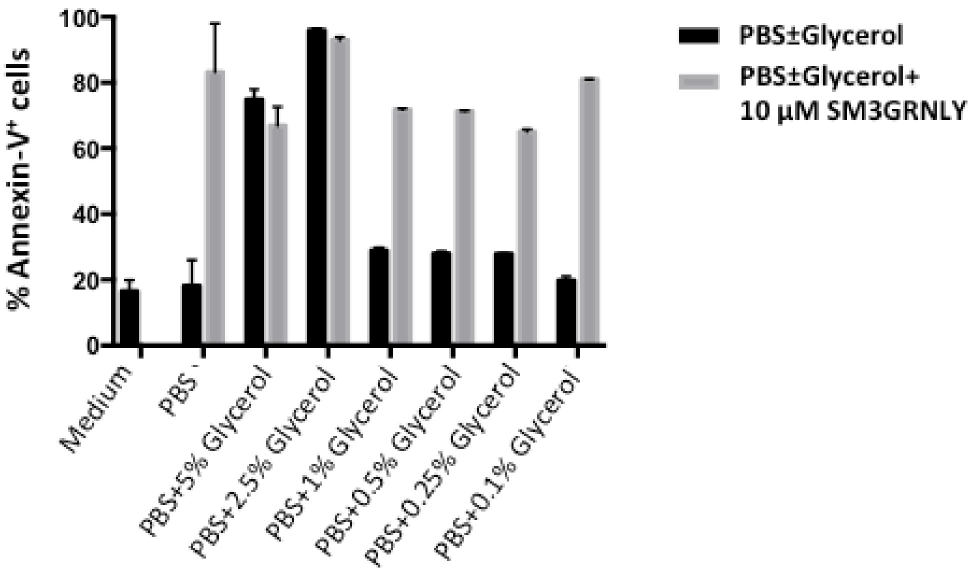

Supplemental Figure 2. Glycerol toxicity tests on Jurkat cells in the presence of SM3GRNLY. Cells were incubated during 24h with the indicated concentrations of glyclerol in PBS, alone (black bars) or in the presence of 10 µM SM3GRNLY (grey bars) and cell death was estimated by Annexin-V-FITC staining and flow cytometry.

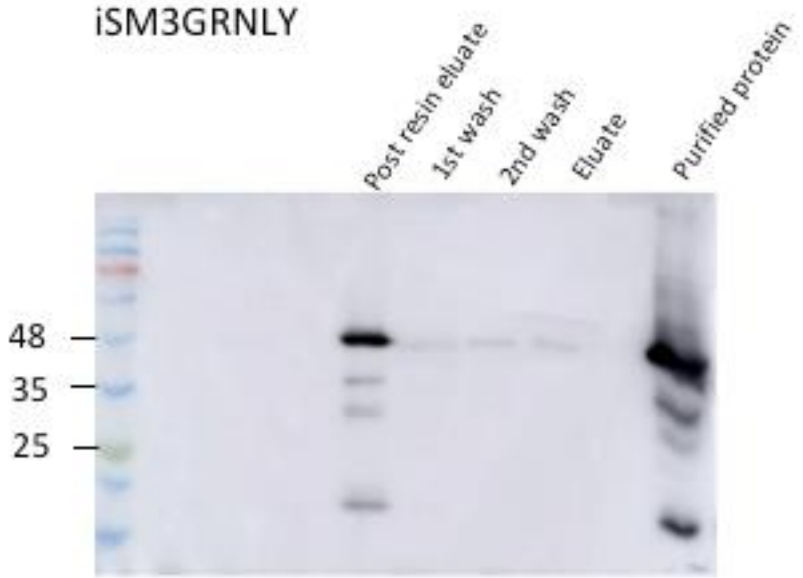

Supplemental Fig. 3. Anti-His inmmunoblot of different steps of the purification process on the Ni-NTA-agarose column of a representative iSM3GRNLY batch obtained using the optizimed PEF method.
